# The Pathology of Old Age

**Published:** 1888-01

**Authors:** 


### THE PATHOLOGY OF OLD AGE.

A work of unusual interest in many respects is one recently published by Dr. Émile Demange, of Paris, upon the pathological anatomy and clinical history of old age. Dr. Demange has had unusual opportunities for studying senility in its various phases in the Hospital for the Aged, at Nancy. His pathological observations include more than five hundred autopsies.

Like most popular French scientists, Dr. Demange delights in reducing things to a formula. In the present case the dominating idea of the book is that the changes of senility are due to disease of the blood-vessels, and that the arteries make us old. In his five hundred cases there was always vascular disease, at least in the smaller vessels. Its characters were a proliferation of the endothelium, thickening of the middle muscular coat, and proliferation of the external coat—in fine, a "fibrous endo-peri-arteritis," tending to narrow and close the vessel's lumen.

The sclerosis thus produced tends to radiate from the blood-vessels, and causes wasting of the parenchyma. Such is the view of Demange.

Charcot, on the other hand, attributes the wasting to insufficient nutrition, through partial closure of the vessels. In the larger vessels atheroma takes place through the obliteration of vasa vasorum by arteritis. In the heart there is first an endo-peri-arteritis of the coronary arteries, consequent sclerosis of the cardiac muscle, atheroma of the endocardium and valves, and finally hypertrophy and dilatation, due to the peripheral obliteration. This compensatory hypertrophy of the senile heart has been described by Charcot and others. The signs of this senile hypertrophy are not always especially characteristic; indeed, in old people the heart is one of the best of the organs. Owing to the atrophied and emphysematous lungs, and to the more rigid thorax, the blood of the aged is poorly oxidized, and has more of a venous character than in earlier life; it coagulates more easily; the corpuscles and the hæmoglobin diminish, while cholesterin and urea increase. The blood is more toxic in quality, therefore.

The central temperature of the aged, contrary to general belief, is not much different from that of early life. The digestive organs are affected in varying degrees with atrophic change. The mucous membrane of the stomach and intestines is white, thin, and glossy, and its arteries atheromatous. The liver is small and sclerotic, while the kidney can hardly be distinguished by the naked eye from the cirrhotic or gouty kidney.

Similar atrophic and fibrous changes affect the system throughout. They are all due primarily, according to Demange, to the vascular disease. This lessens the nutrition, the cells atrophy, sclerosis follows, or, still more often, the sclerosis radiates from the vessels and encroaches on the parenchyma, compressing the cells and causing their atrophy.

To the question, What causes the arterial disease? Demange answers that it is the irritant effect of an excess of products of dis-assimilation circulating in the blood, a vice of metabolism. But as this excess of irritants comes from the tissue-cells, we find that senility, after all, is due to that loss of balance between assimilation and dis-assimilation which is part of the great biological law of life and growth. We must grow old; it is registered in the protoplasm of the cell from the moment that life was imparted to it.—*The Medical Record.*

